# Cerebral Syphilitic Gumma within 5 Months of Syphilis in HIV-Infected Patient

**DOI:** 10.3201/eid2210.160600

**Published:** 2016-10

**Authors:** Motoyuki Tsuboi, Takeshi Nishijima, Katsuji Teruya, Yoshimi Kikuchi, Hiroyuki Gatanaga, Shinichi Oka

**Affiliations:** National Center for Global Health and Medicine, Tokyo, Japan (M. Tsuboi, T. Nishijima, K. Teruya, Y. Kikuchi, H. Gatanaga, S. Oka);; Kumamoto University, Kumamoto, Japan (H. Gatanaga, S. Oka)

**Keywords:** cerebral syphilitic gumma, syphilis, neurosyphilis, Treponema pallidum, bacteria, HIV/AIDS and other retroviruses, viruses, computed tomography, magnetic resonance imaging

**To the Editor:** Tertiary syphilis, including cerebral syphilitic gumma, usually occurs >10 years after contracting syphilis (*1*) and is a rare manifestation since the introduction of penicillin (*2*). However, progression of syphilis is reported to be faster in HIV-infected patients than in those without such infections (*3*). We report a case of cerebral syphilitic gumma in an HIV-1–infected patient for whom serum samples obtained as recently as 5 months earlier showed negative results for syphilis.

A 21-year-old man infected with HIV came to the AIDS Clinical Center, National Center for Global Health and Medicine (Tokyo, Japan), because of a 2-hour loss of consciousness. He reported an uncomfortable feeling at the back of his head and neck and eye fatigue that lasted for 1 week. His HIV-1 infection was well-controlled with an antiretroviral combination of tenofovir, emtricitabine, and dolutegravir. The patient had a CD4 count of 565 cells/μL and a viremia level below detectable limits (<20 copies/mL). He was not using any other medications. 

At examination, his vital signs were within reference ranges. Apart from a tongue bite, physical and neurologic examinations showed no abnormal findings. Results for chest radiograph, Holter electrocardiogram, and electroencephalogram were unremarkable. There were no abnormal ophthalmologic findings. Computed tomography of the brain showed a hypodense lesion at the left frontal lobe (Figure, panel A). Subsequent magnetic resonance imaging showed that the lesion (mass) was hypointense by gadolinium-enhanced, axial, T1-weighted imaging (Figure, panel B), hyperintense by T2-weighted imaging, and surrounded by extensive cerebral edema (Figure, panel C).

**Figure:**
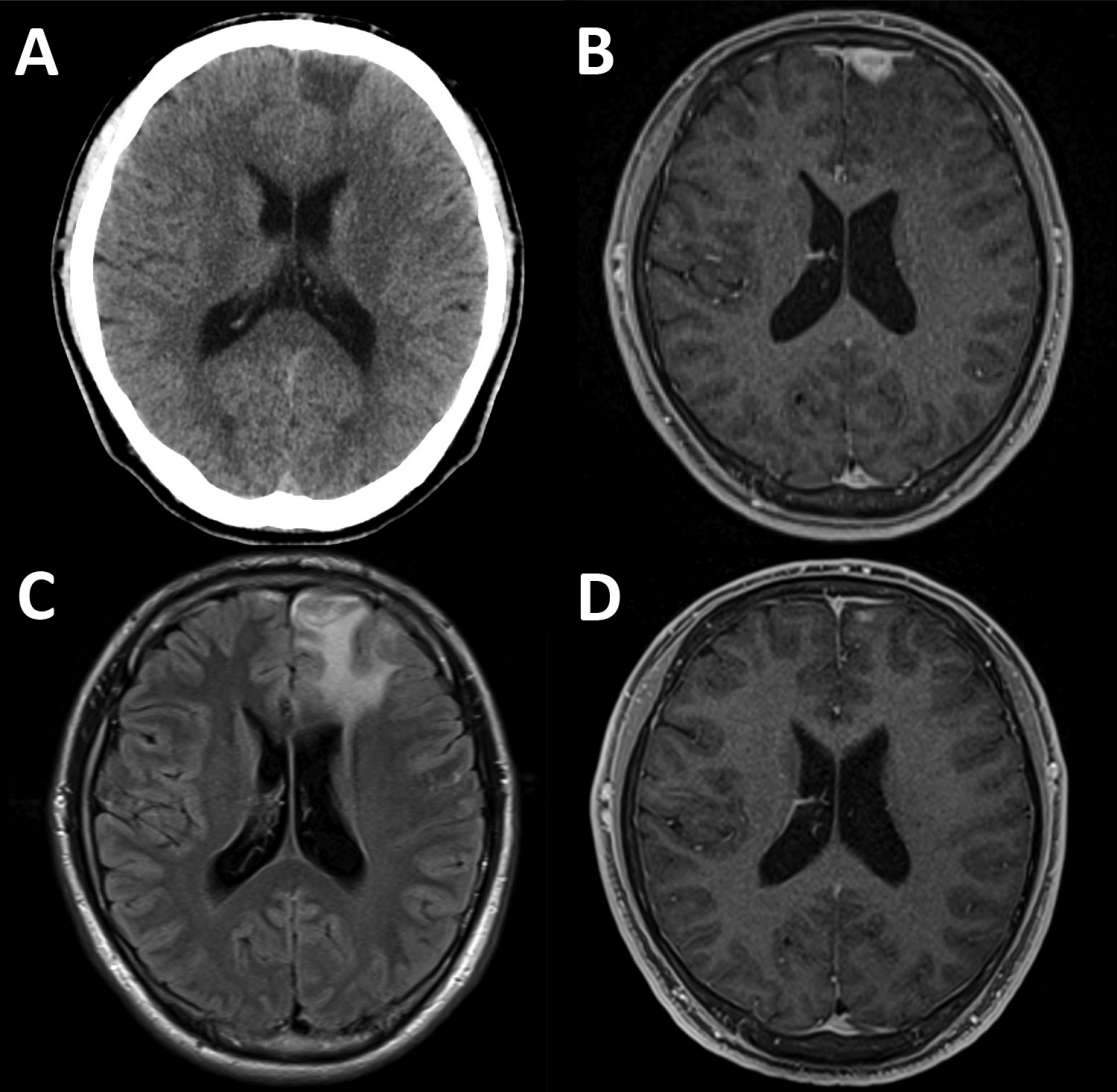
Diagnostic imaging results for a brain mass in a 21-year-old HIV-positive man with cerebral syphilitic gumma in Tokyo, Japan, for whom serum samples obtained as recently as 5 months earlier showed negative results for syphilis. A) Noncontrast, cranial computed tomography showing a hypodense lesion in the left frontal lobe. B) Gadolinium-enhanced, axial, T1-weighted magnetic resonance imaging (MRI) showing an enhanced lesion (mass) (14 × 14 × 12 mm) adjacent to the enhanced dura in the left frontal lobe. C) Axial, fluid-attenuated inversion recovery MRI showing extensive left frontal edema. D) Two months after treatment for syphilis, gadolinium-enhanced, axial T1-weighted MRI showing substantial resolution of the lesion.

Symptomatic epilepsy caused by the mass was suspected to have caused the loss of consciousness. This conclusion was based on the intracranial mass, long duration of loss of consciousness, increase in creatine kinase level (471 U/L), and tongue bite.

When the HIV-1 infection was diagnosed in the patient 15 months earlier, results of serum rapid plasma reagin (RPR) and *Treponema pallidum* hemagglutination test (TPHA) were negative. However, during this examination, serum RPR and TPHA titers were 1:32 and 1:10,240, respectively. Results of cerebrospinal fluid (CSF) analysis were compatible with neurosyphilis (*4**,**5*) and showed a leukocyte count of 35 cells/μL (2 neutrophils/μL, 33 lymphocytes/μL), a total protein level of 30 mg/dL, a glucose level of 59 mg/dL (serum glucose level 92 mg/dL), an RPR titer of 1:<1, a TPHA titer of 1:160, and a fluorescent treponemal antibody-absorption titer of 1:32.

Cerebral syphilitic gumma was suspected on the basis of neurosyphilis and compatible imaging findings (*6*) and because other conditions, such as meningioma, primary central nervous system lymphoma, toxoplasmosis, cryptococcoma, tuberculoma, or brain abscess, were unlikely. These results were based on radiologic findings; high CD4 cell counts; and negative results for Epstein-Barr virus and malignant cells in CSF; serum cryptococcal antigen; CSF culture for bacteria, mycobacteria, and fungi; and interferon-γ release assay.

We did not perform a biopsy for the patient because of presumed high pretest probability of cerebral syphilitic gumma, the invasiveness of this complication, and the young age of the patient. However, successful therapy confirmed the diagnosis (*3*).

We empirically treated the patient with intravenous benzylpenicillin (24 million units/d for 14 consecutive days). Clinical symptoms improved shortly after treatment. At 2-month follow-up, all clinical symptoms and signs had resolved, and a 4-fold decrease in RPR titer was observed (RPR titer 1:8, TPHA titer 1:5,120) (*7*). The brain mass was substantially reduced, which confirmed the diagnosis of cerebral syphilitic gumma (Figure, panel D).

Gumma is a complication of long-term infection with *T. pallidum*, which develops 1–46 years after healing of secondary lesions; most cases develop within 15 years (*1*). However, for our patient, cerebral syphilitic gumma developed within 5 months after he contracted syphilis. After written informed consent was obtained, serum samples were obtained from the patient at his first and subsequent clinic visits and stored. Samples obtained at 11 months, 10 months, 9 months, 5 months, 11 weeks, and 5 weeks before detection of the brain mass were then tested retrospectively for RPR and TPHA titers. Results were negative at 11, 10, 9, and 5 months before detection of the brain mass, but TPHA titers became positive (1:80) at 11 weeks before presentation, and RPR titers became positive (1:16) at 5 weeks before presentation. Because RPR and TPHA titers can become positive as late as 6 weeks after infection (*8*), we believe that the patient contracted syphilis within 5 months before documentation of the cerebral mass.

In conclusion, we report an HIV-1–infected patient in whom cerebral syphilitic gumma developed within 5 months after contracting syphilis. Cerebral syphilitic gumma should be considered in the differential diagnosis of a cerebral lesion in sexually active patients even if they had recently contracted syphilis. Moreover, as guidelines recommend (*9*), screening of HIV-infected patients who are sexually active with multiple partners should be conducted every 3–6 months for early detection of syphilis and initiation of proper treatment to prevent transmission and progression to late syphilis.
